# Clinical application of superselective transarterial embolization of renal tumors in zero ischaemia robotic-assisted laparoscopic partial nephrectomy

**DOI:** 10.3389/fonc.2023.1212696

**Published:** 2023-08-22

**Authors:** Haichang Li, Yuning Hu, Dongning Lu, Jingyun Wang, Yanze Lin, Xugang Zhong, Yixuan Mou, Cenchao Yao, Zhida Wang, Xinyu Zhang, Qijun Wo, Hanbo Liu, Feng Liu, Dahong Zhang, Heng Wang

**Affiliations:** ^1^ Urology & Nephrology Center, Department of Urology, Zhejiang Provincial People’s Hospital (Affiliated People’s Hospital, Hangzhou Medical College), Hangzhou, Zhejiang, China; ^2^ Cancer Center, Department of Interventional Medicine, Zhejiang Provincial People’s Hospital, Affiliated People’s Hospital, Hangzhou Medical College, Hangzhou, China

**Keywords:** superselective renal artery embolization, zero ischemia, partial nephrectomy, nephron-sparing surgery (NSS), robotic-assisted

## Abstract

**Objective:**

To assess the feasibility and safety of zero ischaemia robotic-assisted laparoscopic partial nephrectomy (RALPN) after preoperative superselective transarterial embolization (STE) of T1 renal cancer.

**Methods:**

We retrospectively analyzed the data of 32 patients who underwent zero ischaemia RALPN after STE and 140 patients who received standard robot-assisted laparoscopic partial nephrectomy (S-RALPN). In addition, we selected 35 patients treated with off-clamp RALPN (O-RALPN) from September 2017 to March 2022 for comparison. STE was performed by the same interventional practitioner, and zero ischaemia laparoscopic partial nephrectomy (LPN) was carried out by experienced surgeon 1-12 hours after STE. The intraoperative data and postoperative complications were recorded. The postoperative renal function, routine urine test, urinary Computed Tomography (CT), and preoperative and postoperative glomerular filtration rate (GFR) data were analyzed.

**Results:**

All operations were completed successfully. There were no cases of conversion to opening and no deaths. The renal arterial trunk was not blocked. No blood transfusions were needed. The mean operation time was 91.5 ± 34.28 minutes. The mean blood loss was 58.59 ± 54.11 ml. No recurrence or metastasis occurred.

**Conclusion:**

For patients with renal tumors, STE of renal tumors in zero ischaemia RALPN can preserve more renal function, and it provides a safe and feasible surgical method.

## Introduction

Kidney cancer ranks among the most prevalent cancers in the urinary system (1), and the majority are renal cell carcinomas (RCCs). For localized RCC, surgery is still the only curative treatment. According to renal function, oncological, and quality of life (QoL) outcomes, localized T1 RCCs are best managed with partial nephrectomy (PN) rather than radical nephrectomy (RN), irrespective of the surgical approach (2). PN has become the reference standard for most T1 and an option in T2 renal cortical masses and RCC (2–4) and T3a tumors with oncological equipoise to RN and functional beneft in select patients (5). In the past, LPN had a steep learning curve, which restricted its use by urologists (6). However, the emergence of robot-assisted laparoscopic partial nephrectomy (RALPN) has been shown to reduce the learning curve (7). This operation requires temporary blockade of the renal artery during surgery, which can cause ischaemia-reperfusion injury in the kidney, and with the extension of renal warm ischaemia time (WIT), it will cause irreversible damage to the remaining renal function. To preserve remaining renal function and reduce blood loss, preoperative STE was first described by Gallucci et al. as an option to perform LPN without hilar clamping (8). Before we attempted to start performing this nephron-sparing surgery (NSS) approach, we assessed the safety and effectiveness of RALPN following STE in patients with clinically T1 renal tumors.

## Materials and methods

### Preoperative work-up

We identified 140 patients from 778 patients with renal tumors who received S-RALPN, and 35 patients who received off-clamp RALPN from September 2017 to October 2021 in our hospital.

The data of 32 cases were obtained from May 2015 to March 2022. Before surgery, all patients underwent routine electrocardiogram, pulmonary function examination, color ultrasound, pulmonary CT, CT angiography (CTA), etc. GFR was recorded. No tumors had spread to the regional lymph nodes and no adrenal metastasis or distant metastasis. In addition, patients with renal vein and inferior caval tumors were excluded. The above combination therapy (STE+RALPN) was used after a definite diagnosis.

The inclusion criteria were as follows: 1. preoperative enhanced CT resulted in a diagnosis of renal cancer; 2. tumor stage (TNM) was below stage T2N0M0; 3. patients had no mental illness and were able to tolerate treatments. The exclusion criteria were as follows: patients who had severe lung or heart disease and were not able to undergo interventional surgery.

### Angiographic technique

**Figure 1:**
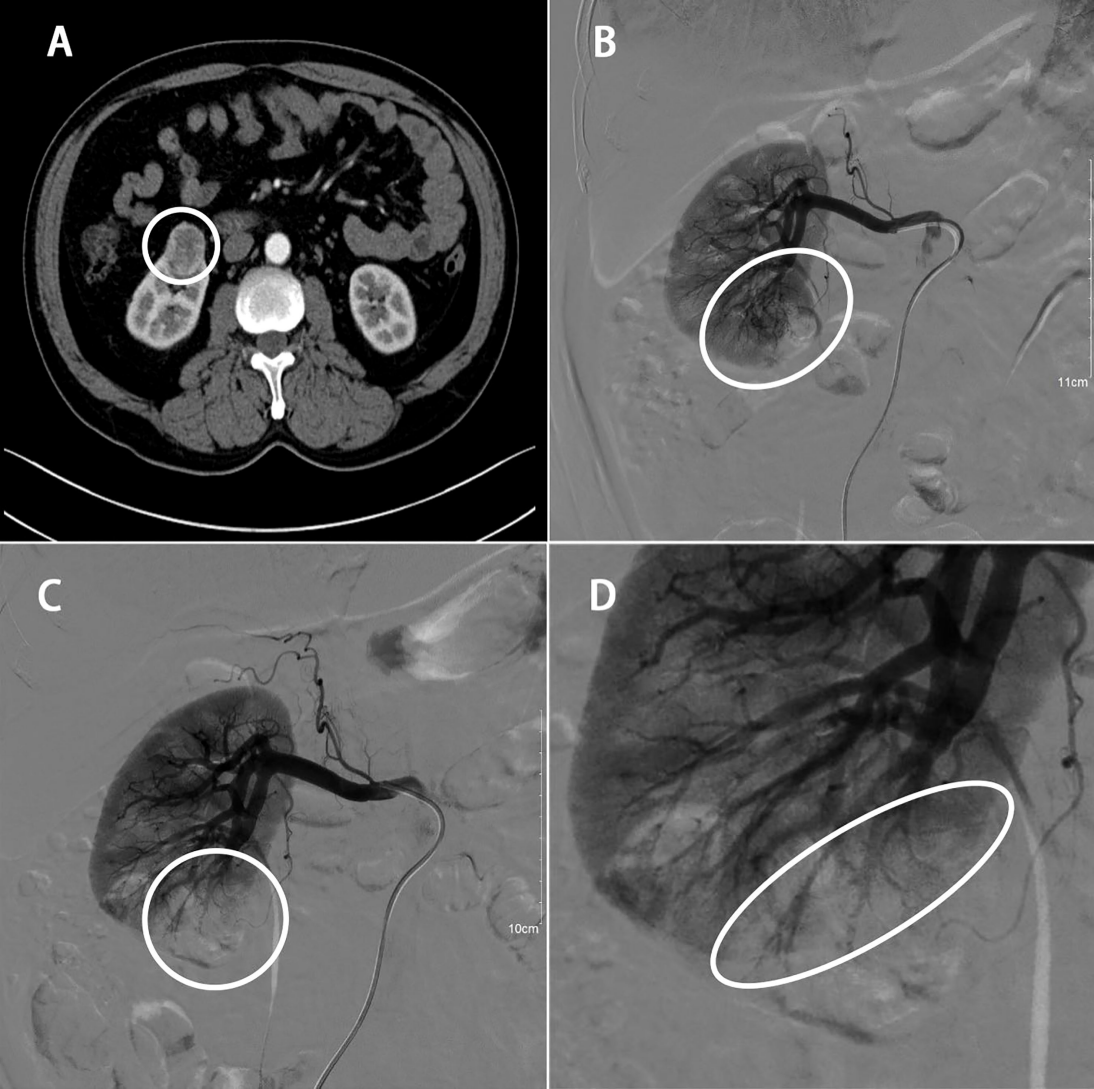
58 years old male with RCC of the right kidney lower pole (40 x 30 x 25 mm). **(A)** Preoperative enhanced CT scan; **(B)** renal arteriogram; **(C)** STE of order artery feeding tumor; **(D)** final angiogram showing appropriate extent of ischemic area.

All patients received prophylactic antibiotic treatment before the initiation of the angiographic procedure, which was completed by a single interventional physician 1-12 h before RALPN. In the supine position, routine disinfection and draping was performed at the groin area. After local anaesthesia with 2% lidocaine, the right femoral artery was punctured with the Seldinger technique and a 5F catheter with its sheath were inserted into the renal artery, and renal artery angiography was performed using a Yashiro catheter. Renal tumor staining was superselected to the renal tumor feeding artery, injected with iodine oil and gelatine sponge particles (100~300 μm), and then a final angiogram was performed to evaluate the adequacy of embolization (**Figure 1**). Disinfection was performed again and a pressure bandage was applied. The surgery was scheduled for the following 1-12 h after embolization.

### Surgical procedure

**Figure 2:**
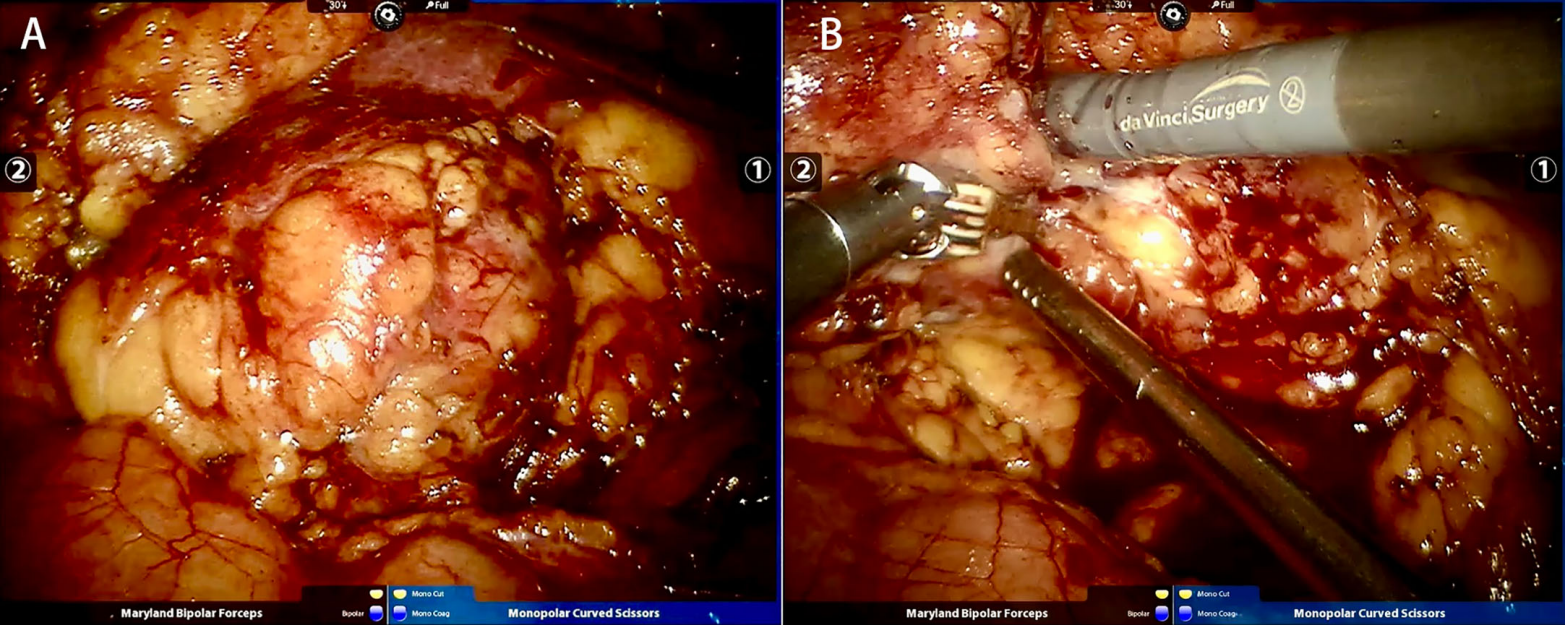
Same case as **Figure 1**. **(A)** Tumor before resection; **(B)** Tumor resection during RALPN.

Taking the right kidney as an example, RALPN was performed 1-12 h following STE. After successful general endotracheal anaesthesia (we needed to maintain a low level of blood pressure), the patient was placed in the left lateral decubitus position. The skin was incised 3 cm superolateral to the umbilicus. This incision was made with a Veress needle to achieve a high flow, low pressure pneumoperitoneum (pressure 15 cm Hg). Then, the needle was replaced with the 12 mm trocar (for camera port). Under visualization, two 8 mm trocars were placed under the costal margin of the midaxillary line and the posterior axillary line. An additional 12 mm port was placed below the two 8 mm robotic ports in the midline, superior or inferior to the umbilicus, for assistance. Prior to renal tumor dissection, the renal hilum (renal artery and vein) was selectively identified and isolated by a combination of blunt dissection and electrocautery. Temporary blockade of the main renal artery was determined by the degree of bleeding during intraoperative tumor resection. The dorsal and posterior sides of the kidney were separated to fully expose the tumor, and a clear oedema band was seen, absorbing the surface blood. The tumor was completely cut at approximately 0. 5 cm from the tumor boundary (**Figure 2**). The wound was sutured with 3-0 and 2-0 absorbable barbed sutures in multiple layers. The specimen was secured into an endobag. After completing haemostasis, a perirenal drainage tube was placed. The specimen was removed, and the incision was closed layer by layer.

### Statistical analysis

SPSS statistical 25.0 was used to process the data. The measurement data in accordance with the normal distribution are expressed by means ± SDs, and the measurement data that do not conform to the normal distribution are expressed by the median (range). The Wilcoxon rank sum test was used to compare the median data. The counting data were expressed by number or percentage (%), and the comparison among the three groups was conducted by an ANOVA test and the exact probability method. The difference was statistically significant if P < 0.05.

## Results

All 207 operations in this study were completed successfully, and none of them resulted in an open or blocked renal artery. The patients’ characteristics are summarized in **Table 1**. The distribution of age, sex, Body Mass Index (BMI), American Society of Anesthesiology (ASA) score, laterality, mass location, and R.E.N.A.L. score were comparable among the three groups. The perioperative and pathologic data are summarized in **Table 2**. Although the surgical position was changed after embolization, there were no STE-related complications. The ages of the STE-RALPN, O-RALPN and S-RALPN groups were 55.88 ± 8.40, 55.11 ± 11.27 and 56.56 ± 12.04 (p> 0.05), respectively. 78.1% of these patients were male, and the ASA score was low (1–2 points; 90.6%). The R.E.N.A.L. score was low (4–6 points; 84.4%) (P=0.001). The mean BMIs of the STE-RALPN, O-RALPN and S-RALPN groups were 22.93 ± 3.13, 24.73 ± 3.50 and 24.56 ± 3.20, respectively (P=0.03).

**Table 1:** Comparison of demographic data (mean ± SD).

Project	STE+RALPN (n=32)	Off-clamp RALPN (n=35)	S-RALPN (n=140)	p value
Age (year), (mean ± SD)	(55.88 ± 8.40)	(55.11 ± 11.27)	(56.56 ± 12.04)	P> 0.05
Gender, n (%)				P> 0.05
Male	25 (78.1)	29 (82.9)	89 (63.6)	
Female	7 (21.9)	6 (17.1)	51 (36.4)	
BMI (kg/m2), (mean ± SD)	(22.93 ± 3.13)	(24.73 ± 3.50)	(24.56 ± 3.20)	P=0.03
ASA score, n (%)				P> 0.05
1	6 (18.7)	3 (8.6)	10 (7.1)	
2	23 (71.9)	30 (85.7)	115 (82.1)	
3	3 (9.4)	2 (5.7)	15 (10.7)	
Tumor size	(3.00 ± 2.08)	(2.34 ± 1.19)	(3.17 ± 1.27)	P=0.009
Laterality, n (%)				P> 0.05
center	19 (59.4)	21 (60)	67 (47.9)	
Right	13 (40.6)	14 (40)	73 (52.1)	
Mass location, n (%)				P> 0.05
Upper	14 (43.7)	14 (40)	50 (35.7)	
Interpolar	11 (34.4)	11 (31.4)	53 (37.9)	
Lower	7(21.9)	10 (47.6)	37 (26.4)	
Renal score, n (%)				P=0.001
Mild complexity (4–6)	27 (84.4)	31 (88.6)	116 (82.9)	
Moderate complexity (7–9)	5 (15.6)	4 (11.4)	24 (17.1)	
High complexity (10–12)	0 (0)	0 (0)	0 (0)	

BMI, Body Mass Index:kg/m2.

**Table 2:** Comparison of perioperative data between two groups (mean ± SD).

Project	STE+RALPN(n=32)	Off-clamp RALPN(n=35)	S-RALPN (n=140)	p value
Operative time, min	(91.59± 34.28)	(96.86 ± 25.87)	(123.29 ± 33.98)	P<0.001
WIT, min	0	0	(19.03 ± 4.53)	P> 0.05
EBL, ml	(58.59 ± 54.11)	(62.86± 34.35)	(96.71± 99.93)	P=0.029
24-h hemoglobin drop down, g/dL	(16.63 ± 7.79)	(16.6 ± 8.79)	(21.11± 12.08)	P=0.016
Drainage tube removal time, d	(4.20 ± 1.34)	(4.63 ± 1.14)	(4.82 ± 1.30)	P=0.059
hospital stay, d	(5.72 ± 1.35)	(5.91 ± 1.22)	(6.05 ± 1.50)	P> 0.05
Intraoperative blood transfusion				P> 0.05
Yes	0 (0)	0 (0)	0 (0)	
No	32 (100)	35 (100)	140(100)	
Postoperative blood transfusion				P> 0.05
Yes	0 (0)	0 (0)	0 (0)	
No	32 (100)	35 (100)	140(100)	
Histopathological results, n (%)				P> 0.05
Clear cell	28 (87.5)	29 (82.8)	128(91.4)	
Angiomyolipoma	2 (6.25)	0 (0)	1(0.7)	
Papillary	2 (6.25)	3(8.6)	4(2.9)	
Chromophobe cell	0 (0)	3(8.6)	7(5)	
Pre-operative GFR/(ml/min)	(86.71 ± 20.02)	(88.72 ± 22.27)	(91.64 ± 19.22)	P> 0.05
1week Post-operative GFR (ml/min)	(85.81 ± 21.36)	(85.31 ± 24.06)	(81.78 ± 21.73)	P> 0.05
GFR decline in 1week	(0.89 ± 11.43)	(3.42 ± 11.01)	(9.86 ± 16.63)	P= 0.03
3 months Post-operative GFR (ml/min)	(84.17 ± 18.32)	(84.72 ± 21.37)	(81.95 ± 19.27)	P> 0.05
GFR decline in 3 months	(2.54 ± 10.08)	(4.00 ± 9.98)	(9.68 ± 15.45)	P= 0.009

GFR, glomerular filtration rate; WIT, Warm ischemia time; EBL, estimated blood loss.

The mean operative times in the STE-RALPN group and O-RALPN group were very similar, half an hour less than that in the RALPN group (P<0.001). The warm ischaemia time only existed in the S-RALPN group, so it was not comparable. The mean estimated blood loss (EBL) was 58.59 ± 54.11, 62.86 ± 34.35 and 96.71 ± 99.93 (P=0.029) in the STE-RALPN, O-RALPN and S-RALPN groups, respectively, with a mean 24-h haemoglobin drop after surgery of 16.63 ± 7.79, 16.6 ± 8.79 and 21.11 ± 12.08 (P=0.016), respectively. Postoperative pathology revealed renal angiomyolipoma in a total of 3 cases (1.45%), and renal clear cell carcinoma was the main pathological type in 163 cases (89.37%) among the three groups. The number of positive surgical margins and blood transfusions was 0 in the three groups. The mean drainage tube removal times were 4.20 ± 1.34, 4.63 ± 1.14 and 4.82 ± 1.30 (P=0.0059) in the STE-RALPN, O-RALPN and S-RALPN groups, respectively. We analyzed the preoperative versus postoperative GFR decline, which was comparable at 1 week and 3 months after the operation. The mean GFR decline 1 week after the operation in STE-RALPN, O-RALPN and S-RALPN was 0.89 ± 11.43, 3.42 ± 11.01 and 9.86 ± 16.63, respectively (P= 0.03), and 3 months after the operation was 2.54 ± 10.08, 4.00 ± 9.98 and 9.68 ± 15.45, respectively(P= 0.009).

## Discussion

Embolization via Digital Subtraction Angiography (DSA) is an auxiliary technique for angiography and embolization by the floating catheter to the target vessel through a superficial arterial approach. Its initial indications were limited to symptomatic haematuria and palliative care for metastatic renal cancer (9). With advances in technology and experience, artery embolization has been widely used to treat numerous conditions as a safe and feasible minimally invasive procedure.

At present, to control intraoperative bleeding and maintain a clear operating field, blocking the renal artery with a hilar clamp has become the standard operating procedure. The advantages of this method are that the intraoperative blood loss is reduced and the operating field is clear (10), which is conducive to accurate cutting and suturing. However, due to the limitation of WIT, the operation time will be limited to less than 30 min to avoid irreversible damage to renal function. It has been reported that approximately 20% of patients have acute renal failure even when the average WIT is less than 30 min, and the incidence of postoperative renal function decline and renal insufficiency in isolated kidney patients will be further increased. In LPN, there is no absolutely safe WIT, and every single minute that the renal artery is blocked causes damage to the kidney (11). Therefore, many scholars have begun to explore different methods to protect renal function when blocking the renal artery, mainly focusing on the cooling of the operation field, including ice or ice water around the kidney and saline perfusion through the renal artery (12–15). However, hypothermia can only appropriately prolong the duration of renal ischaemia, and studies have shown that patients with cold ischaemic LPN can tolerate ischaemia for a longer time (45 min and 22 min, respectively). Therefore, complete unblocked nephrectomy of the renal artery is necessary.

STE-RALPN has many advantages. First, it can avoid WIT and its associated risk of acute tubular necrosis. Second, there is no need to isolate the renal artery in every patient, especially in patients whose renal artery is too difficult to explore because of anatomical abnormalities or previous inflammation. Third, the complication rate of renal vessel injury is reduced (16). In addition, this current research is based on the assistance of the Da Vinci robot SI, which represents a developing and innovative area in many surgical specialties, including urology (17). It could offer a better field of operation (3D magnified vision) to the surgeon and provide a more precise and smart operation to reduce the amount of intraoperative blood loss and less tissue contact to improve infection risk and protect renal function after surgery than traditional LPN (18). We can conclude that the robotic-assisted procedure was an independent protective factor and offered a lower rate of complications.

However, since S-RALPN has a learning curve requiring 30-40 cases to master (19) and selectively identify and isolate the renal hilum, especially for surgeons at the beginning of their career in robotic surgery, RAPN may lead to suboptimal outcomes. STE via DSA perfectly solves the problem. STE before surgery was first described by Gallucci et al. (8) as an option in partial nephrectomy without renal artery blockade, and subsequent studies consistently show that this technique results in a lower rate of postoperation complications without the limitation of WIT.

In 2013, a concept of trifecta outcomes was introduced by Gallucci et al. (20) during RALPN/LPN, in which the 3 key outcomes of negative cancer margin, minimal renal functional decrease and no urological complications are simultaneously realized (21). Recently, “Pentafecta” was defined by Sri et al. (22) as achievement of “Trifecta” (negative surgical margin, no postoperative complications and WIT of < 25 min) plus over 90% GFR preservation and no Chronic kidney disease (CKD) stage upgrading at 1 year. We believe that from the above standards, the results of our experiment are relatively successful. We have achieved negative surgical margins, no complications, WIT < 25min, and postoperative preservation of more than 90% of renal function on “Pentafecta”. In addition, we do not discover CKD in patients after operation, and the probability of developing CKD in one year is not high without the interference of other diseases.

Even if STE can reduce blood loss and renal function impairment (23), we must keep in mind the potential complications of STE. Possible complications include puncture site bleeding, embolus from vessel atheroma, infarction and secondary hypertension. However, none of the above complications were observed in our patients (16). Only one of the cases had postoperative bowel obstruction due to a colonic diverticulum. We attribute the good results without interventional complications to the experienced interventional radiologist who performed STE.

Although STE-RALPN resulted in a loss of a portion of the renal parenchyma due to embolism, the mean GFR decline was larger for the off-clamp RALPN group and much larger for the S-RALPN Group 1 week after the operation. Hence, these results reveal that STE-RALPN is more effective than off-clamp RALPN and S-RALPN(P= 0.03). Although statistical significance was not reached, it must be pointed out that patients who received STE-RALPN were younger with a slightly higher preoperation GFR. For 24-hour haemoglobin loss and EBL, there was no significant difference between STE-RALPN group and off-clamp RALPN group, but it’s higher in S-RALPN group. Another study by D’Urso et al. (23) using STE+LPN reported similar therapeutic effects.

There are also different voices in this field. Abdel Raheem et al. published a funding shows that WIT lengths during PN has no effect on the long-term renal function outcomes in patients having two kidneys and preoperative eGFR≥60 mL/min/1.73m2 (24). We believe that under certain conditions WIT may have little effect on renal function in patients as these authors suggest. But in our perspective, for older patients, patients with diabetes and other groups, every minute of WIT has a significant impact on residual kidney function. Moreover, Chung et al. reported short WIT was not associated with better postoperative kidney function or survival after PN in patients with stage III CKD (25). This paper is similar to the finding published by Hakmin Lee et al. Lee said prolonged WIT was not associated with increased incidence of CKD or MRFD (major renal function deterioration; defined as an eGFR decrease of ≥25% postoperatively)after PN (26). These publications challenge the theory that ischaemic nephropathy is inevitable if the renal vessels are clamped beyond 30 min, leading to a long-term decline in renal function. It seems to make sense that kidney function depends on the quantity and quality of renal parenchyma and is not directly related to WIT length. Although WIT can cause damage to kidney parenchyma and decrease in kidney function, it is not a linear relationship. It is hard to ignore such data in exchange for this a contemporary study when so much is at stake for the patients’ renal function. The type and duration of ischaemia remain the most important modifiable factors, we just need to shorten WIT. Clearly, factors other than WIT contribute to postoperative renal function but for now we must conclude that every minute ‘on-clamp’ does count.

As for elderly patients and those with comorbidities or solitary kidney, including Completely endophytic renal masses, these who are not ideal surgical candidates and NSS is crucial.In the past several years,Percutaneous tumor ablation (PTA) is recognized by guidelines as a safe NSS option, especially for unfit surgical candidates. Recently, Pandolfo et al.reported some multi-institutional mature experience that PTA confirms to be an effective treatment for completely endophytic renal masses, offering low complications and good mid-term functional and oncologic outcomes (27, 28). PTA can be safely offered as treatment option in this challenging clinical scenario, nevertheless, the studies of Pandolfo et al. and Bianchi et al. showed that compared with RALPN, PTA might carry a higher risk of recurrence (29). The data supporting the safety of ablation therapy were limited by the heterogeneity of the study cohort, selection bias, lack of long-term follow-up data, etc. Therefore, trials with standardized reporting, extended follow-up periods, and prospective studies are needed to further determine the role of ablation techniques in the clinical practice of small renal masses.

This is a retrospective study with limitations due to sample size and the lack of long-term renal function measurements and late oncologic outcomes in our study. We will continue to increase the number of experimental subjects and continue to follow up postoperative patients in the future. The results of this study provide support for our hypotheses, but in the future, we will need larger sample sizes and follow-up data to confirm our findings. STE is not the standard surgical method, and consequently we were unable to select the subjects randomly due to the cost that includes the cost of interventional embolization and the cost of robotic surgery. With the expansion of health care programs and commercial insurance, we believe these things will work out over time.

## Conclusion

The STE technique based on angiography and embolization by the floating catheter is a relatively safe and effective preoperative embolization technique. The main advantages of preoperative embolization of the renal artery are as follows. This surgical technique cuts off tumor vascular supply without the use of WIT, which results in a low intraoperative blood loss and lower risk of postoperative complications. Additionally, the use of the STE technique can help the surgeon complete the learning curve smoothly. We believe that STE+RALPN in stage T1 renal cancer is safe and feasible, especially for patients with RCC combined with solitary kidney renal inadequacy and potential risk for decreased renal function.

## Data availability statement

The original contributions presented in the study are included in the article/supplementary material. Further inquiries can be directed to the corresponding authors.

## Ethics statement

The studies involving human participants were reviewed and approved by the ethics committee of Zhejiang Provincial People’s Hospital (KY2022015). The patients/participants provided their written informed consent to participate in this study.

## Author contributions

HCL, HW, and YH: Data collection, Data analysis, Manuscript writing. YL, DL, XYZ, CY, XGZ, ZW, and YM: Data analysis. DZ, HBL, JW, QW, and FL: Project development. HW and HCL: Critical revision of manuscript. All authors contributed to the article and approved the submitted version.
